# Divergent connectomic organization delineates genetic evolutionary traits in the human brain

**DOI:** 10.1038/s41598-021-99082-6

**Published:** 2021-10-04

**Authors:** Elisenda Bueichekú, Jose M. Gonzalez-de-Echavarri, Laura Ortiz-Teran, Victor Montal, Federico d’Oleire Uquillas, Lola De Marcos, William Orwig, Chan-Mi Kim, Elena Ortiz-Teran, Silvia Basaia, Ibai Diez, Jorge Sepulcre

**Affiliations:** 1 Gordon Center for Medical Imaging, Department of Radiology, Massachusetts General Hospital, Harvard Medical School, Boston, USA; 2 Barcelona βeta Brain Research Center, Barcelona, Spain; 3 Department of Radiology, Division of Nuclear Medicine and Molecular Imaging, Brigham and Women’s Hospital, Harvard Medical School, Boston, USA; 4 Memory Unit, Department of Neurology, Hospital de la Santa Creu i Sant Pau, Biomedical Research Institute Sant Pau, Universitat Autonoma de Barcelona, Barcelona, Spain; 5 Centro de Investigacón Biomédica en Red de Enfermedades Neurodegenerativas (CIBERNED), Madrid, Spain; 6 Princeton Neuroscience Institute, Princeton University, Princeton, USA; 7 University of Navarra School of Medicine, University of Navarra, Pamplona, Navarra Spain; 8 Athinoula A. Martinos Center for Biomedical Imaging, Department of Radiology, Massachusetts General Hospital, Harvard Medical School, Charlestown, USA; 9 Facultad de Ciencias Jurídicas y Sociales, Universidad Rey Juan Carlos, Madrid, Spain; 10 Neuroimaging Research Unit, San Raffaele Scientific Institute, Vita-Salute San Raffaele University, Milan, Italy

**Keywords:** Neuroscience, Cognitive neuroscience, Genetics of the nervous system

## Abstract

The relationship between human brain connectomics and genetic evolutionary traits remains elusive due to the inherent challenges in combining complex associations within cerebral tissue. In this study, insights are provided about the relationship between connectomics, gene expression and divergent evolutionary pathways from non-human primates to humans. Using in vivo human brain resting-state data, we detected two co-existing idiosyncratic functional systems: the segregation network, in charge of module specialization, and the integration network, responsible for information flow. Their topology was approximated to whole-brain genetic expression (Allen Human Brain Atlas) and the co-localization patterns yielded that neuron communication functionalities—linked to *Neuron Projection*—were overrepresented cell traits. Homologue-orthologue comparisons using dN/dS-ratios bridged the gap between neurogenetic outcomes and biological data, summarizing the known evolutionary divergent pathways within the *Homo Sapiens* lineage. Evidence suggests that a crosstalk between functional specialization and information flow reflects putative biological qualities of brain architecture, such as neurite cellular functions like axonal or dendrite processes, hypothesized to have been selectively conserved in the species through positive selection. These findings expand our understanding of human brain function and unveil aspects of our cognitive trajectory in relation to our simian ancestors previously left unexplored.

## Introduction

Human beings display a broad variety of cognitive and behavioral features that make us exceptional among primates. Due to this distinctive psychobiological profile, the similarities and differences between human and non-human primate brains have been studied extensively^1–8^. Compared to non-human primates, the human brain has undergone numerous biological changes in the span of the last five to seven million years^8^. On one hand, biological similarities have been found between humans and non-human primates in terms of their genetic code and their molecular and cellular features^9^. Conversely, several investigations have provided evidence for a divergent evolutionary pathway that humans took with respect to non-primates, leading to advanced cognitive features and distinct behaviors^10^. However, the detailed relationships between basic human brain connectomics and its genetic evolutionary traits remain elusive due to the challenges in combining complex associations within the cerebral tissue. In the present work, we contribute new insights to these challenges by investigating the link between the human functional connectome and genome features of the *Homo Sapiens* lineage compared to seven other non-human primates, using a recently developed graph-analytical approach that explores the segregation and integration properties of the brain.

Some other studies posit that the brain prioritizes a state of optimum information processing flow by balancing the amount of segregation and integration within its functional modules^11–13^. It seems that the brain is in a state of dynamic adaptation of these functional modules, which can be measurable by means of functional connectivity. Thus, first aim involved investigating functional connectivity organization patterns or the way discrete groups of neurons communicate composing segregated modules and, in turn, tracking in time the interactions between modules or the integration processes that lead to sensory, motor and cognitive systems. From a broader perspective the segregation and integration processes within the whole brain’s functional connectivity network—simultaneously incorporating novel information into current processing streams while not disrupting the system at the overall organization and energy levels—could have been pivotal for the survival of the species. Yet, the question of the biological relationship between this sophisticated system and the evolution of the *Homo Sapiens* species remains elusive. The combination of connectomics and genetics present the opportunity to explain this relationship from a functional and evolutionary perspective.

Recent research in humans has successfully linked cortical gene expression and neuroimaging connectivity data across a variety of topics^14–31^. Additionally, discovering the similarity between small-world networks and the brain as a complex network system^32–34^ has been a major advancement for better understanding brain systems functioning. This discovery led to the characterization of network properties using graph-based analytical approaches (e.g.,^35–38^). Moreover, graph theory principles have helped advance the field of cognitive neuroscience to formalize connectivity principles^28,39^, making it possible to quantitatively define its hierarchical spatial organization and temporal dynamics^25,40,41^. Furthermore, resources such as the Allen Human Brain Atlas (AHBA^42^) have presented new possibilities to link neuroimaging phenotypes and in situ brain genetic information, offering whole-brain genome-wide expression patterns^27,42^.

Here we have incorporated our connectomic-genetics integration approach (see “Methods” section in^16,25,43^) to measure genetic adaptation across years of evolution, namely, the dN/dS ratio. The dN/dS ratio (or K_a_/K_s_ ratio) specifically quantifies biological selection by taking into account the rate of substitutions in silent and non-silent sites of protein sequences (e.g.,^44^). In this study, we propose the use of a recently developed graph-based analytical method, *merging trajectory analysis toward minimal graphs*, with the purpose of evaluating the different possible connectivity tendencies of a brain voxel, namely segregation or integration. We take one step further by relating the distinctive spatiotemporal functional networks to the evolution of the human brain by linking the connectomics information to transcriptional gene expression provided by the AHBA. This approach let us broad our understanding of human brain function, linking our advanced cerebral connectivity features to our evolutionary history from a biological perspective.

## Results

### Divergent trajectories of segregation and integration connectivity in the human brain

**Figure 1:**
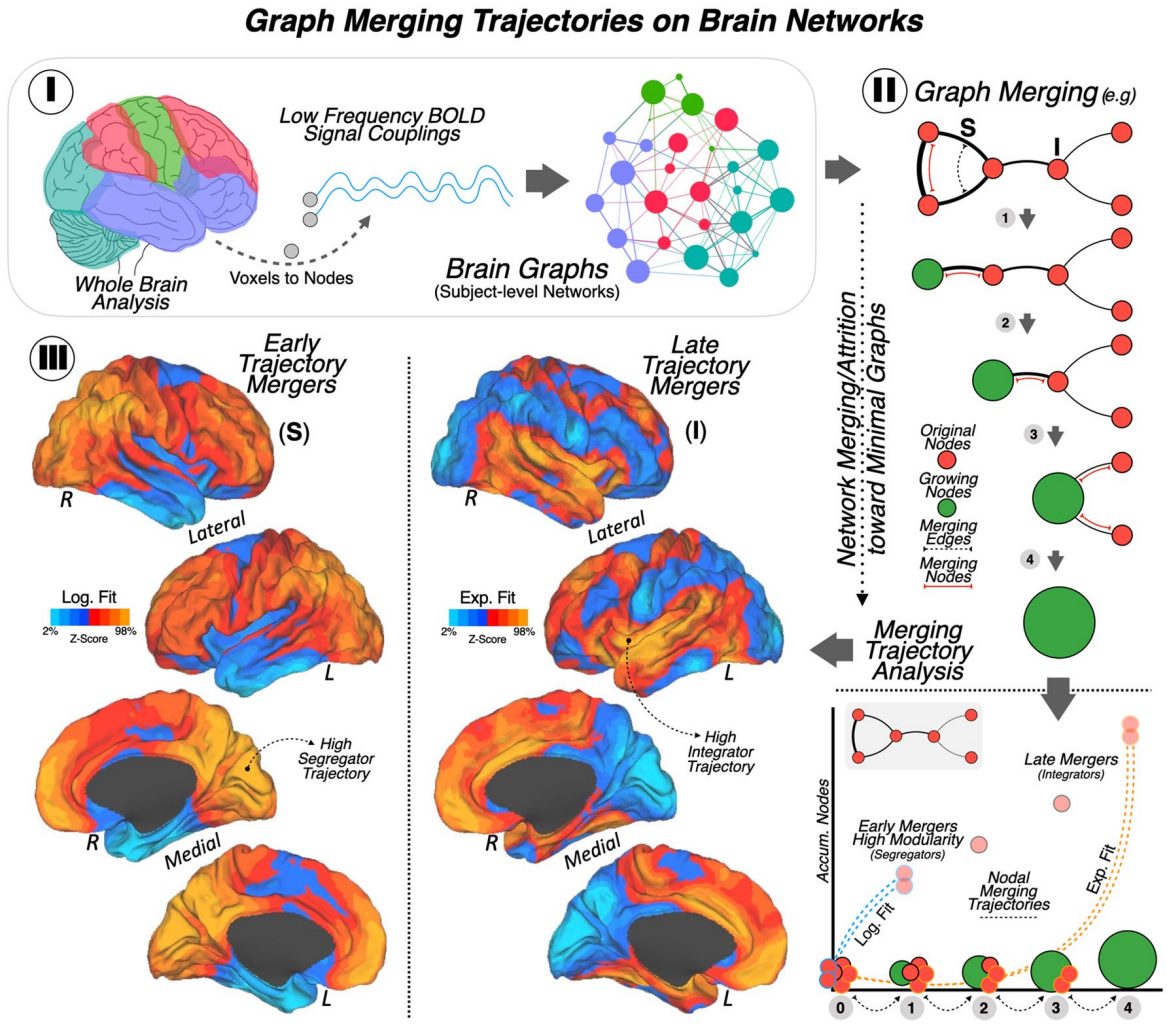
Graph merging trajectories on brain networks (I) Neuroimaging data. Whole-brain functional MRI data measured as low frequency blood oxygenation level-dependent signal couplings of the cerebral cortex were recorded at the voxel level. Graph functional connectivity analysis at the node level was conducted, obtaining connectivity networks at the subject level. (II) Connectomics analyses. An example of our graph merging analysis approach is depicted (upper part in II), illustrating spatiotemporal organization changes in the brain functional networks. (1) Brain nodes, which are prone to be segregated into locally interconnected subsystems (*segregating nodes* or *S*), (2) progressively blend with other discrete nodes by means of interacting with the merging nodes (*integrating nodes* or I), (3) until integrating themselves into complex systems (i.e., *growing nodes*), (4) and finally result in organized large-scale networks or brain systems. The merging trajectory analysis (lower part in II) allows for the investigation of the reorganization of the human brain functional networks from a more segregated to a more integrated state. This analysis was based on implementing logarithmic and exponential curve fitting models after investigating the cumulative node-to-node relations, making it possible to differentiate brain regions prone to merge earlier from those likely to blend later. (III) Topological representations. Brain maps projections reflecting the networks’ spatial distribution after applying the graph merging trajectory analysis to the functional connectivity human brain data: on the left, the early trajectory mergers or the *segregating nodes* were discriminated after implementing a logarithmic curve fitting model; on the right, the late trajectory mergers or the *integrating nodes* were differentiated from the early ones after using an exponential curve fitting model. *Note*: All the individual and group-level analyses were done adopting a whole-brain voxel-level approach and all of them were corrected for multiple comparisons using a False Discovery Rate approach (voxel-wise FDR *q* < 0.05). *BOLD* Blood oxygenation level-dependent, *S* Segregating node, *I* Integrating node, *Log. Fit* Logarithmic curve fitting model, *Exp. Fit* Exponential curve fitting model, *R* Right hemisphere, *L* Left hemisphere.

The objective of the connectomics analysis was to investigate the spatiotemporal configuration of the human brain connectome as minimal graphs. Keeping in mind the brain connectome principles—efficiency maximization and energy cost minimization—we aimed to capture its segregating and integrating properties. Our voxel-wise *merging trajectory analysis* approach as applied to resting-state fMRI data (see Fig. 1-I) made it possible to synthesize the spatiotemporal functional connectivity network properties of the human brain at the cortical level (see Fig. 1-II). The main result yielded two separate patterns of functional connectivity organization (see Fig. 1-III). A logarithmic fit function was used to model the topology of the segregation connectivity network. We observed hubs within the visual and association cortices, and the network extended over lateral and medial parietal cortex (including the precuneus and posterior cingulate cortex), the temporoparietal area, and the lateral frontal cortex (*e.g.,* dorsolateral prefrontal cortex) with some medial frontal regions, especially the frontal pole (see brain maps on the left side of Fig. 1-III). An exponential fit function was used to model the topology of the integration connectivity network. This consisted of nodes that merged later into the functional network, such as posterior to anterior insular cortex, anterior cingulate cortex, temporal cortex (especially the temporal pole), and the medial orbitofrontal cortex (see brain maps on the right side of Fig. 1-III).

We further explored the consistency of our maps and main connectomics results by using two different approaches. We first applied a correction for multiple comparisons to the obtained merging trajectory analysis results. We projected the results without any threshold, i.e., uncorrected maps, as well as with an FDR-correction at *q* < 0.05, *q* < 0.001 and *q* < 0.0001^45^ (see Supplementary Fig. 1). We then entirely repeated the merging trajectory analysis in functional MRI data from an independent sample (see Supplementary Fig. 2). Both validation approaches yielded results that confirmed the original observations. All these results were projected using an FDR-correction *q* < 0.001.

### Intersection between human brain connectomics, genetics and evolution

We used AHBA to investigate the spatial intersection of gene expression data with topological profiles associated with segregation and integration of functional connectivity in the human brain (see Fig. 2; for the purpose of exemplifying this method the relationship between the mean cortical expression of the *Neuron projection* and the functional network map have been represented in Fig. 2-III). A spatial similarity analysis approach allowed us to compare the entire cortical transcriptome of 20,737 genes from the AHBA, distributed across 68 different cortical regions from the Desikan-Killiany atlas^46^, with the mean connectivity segregation—integration map obtained in the previous analytical step. From this analysis, we obtained a null hypothesis distribution of gene expression levels from the AHBA. In the lower-bound (-1.96 SD), we identified 573 genes with transcriptomic expression associated with the topology of the brain areas related to the early mergers or segregating nodes. Similarly, in the upper-bound (+ 1.96 SD), we identified 400 genes with transcriptomic expression associated with the late mergers or integrating nodes (see Fig. 2-I; the list of genes associated with the lower or upper bound appear in Supplementary materials Tables 1 and 2). The specific cellular functional components of genes were identified using Gene Ontology (FDR-corrected q < 0.05); see in Fig. 2-II; the list of cellular components linked to the lower or the upper bound of the probability distribution, and the list of genes associated with each cellular component appear in Supplementary materials Tables 3, 4, 5 and 6. Bearing in mind the purpose of this work, after conducting a curve fitting estimation analysis to relate the connectomics-genetics profiles to evolutionary aspects, we found that the functionalities related to neuron communication were the most relevant results, for example, *Neuron Projection* or *Synapse*, that are closely related to axonal and dendrite processes. As a mean subtraction functional connectivity map was used to draw associations between connectomics and genetics, the genetic expression of the neuronal communication processes involved in the *Neuron projection* would be positively related to brain areas linked to integration, while less expression of these genes is found in modular or highly segregated areas (see Supplementary materials Table 7).

**﻿Figure 2:**
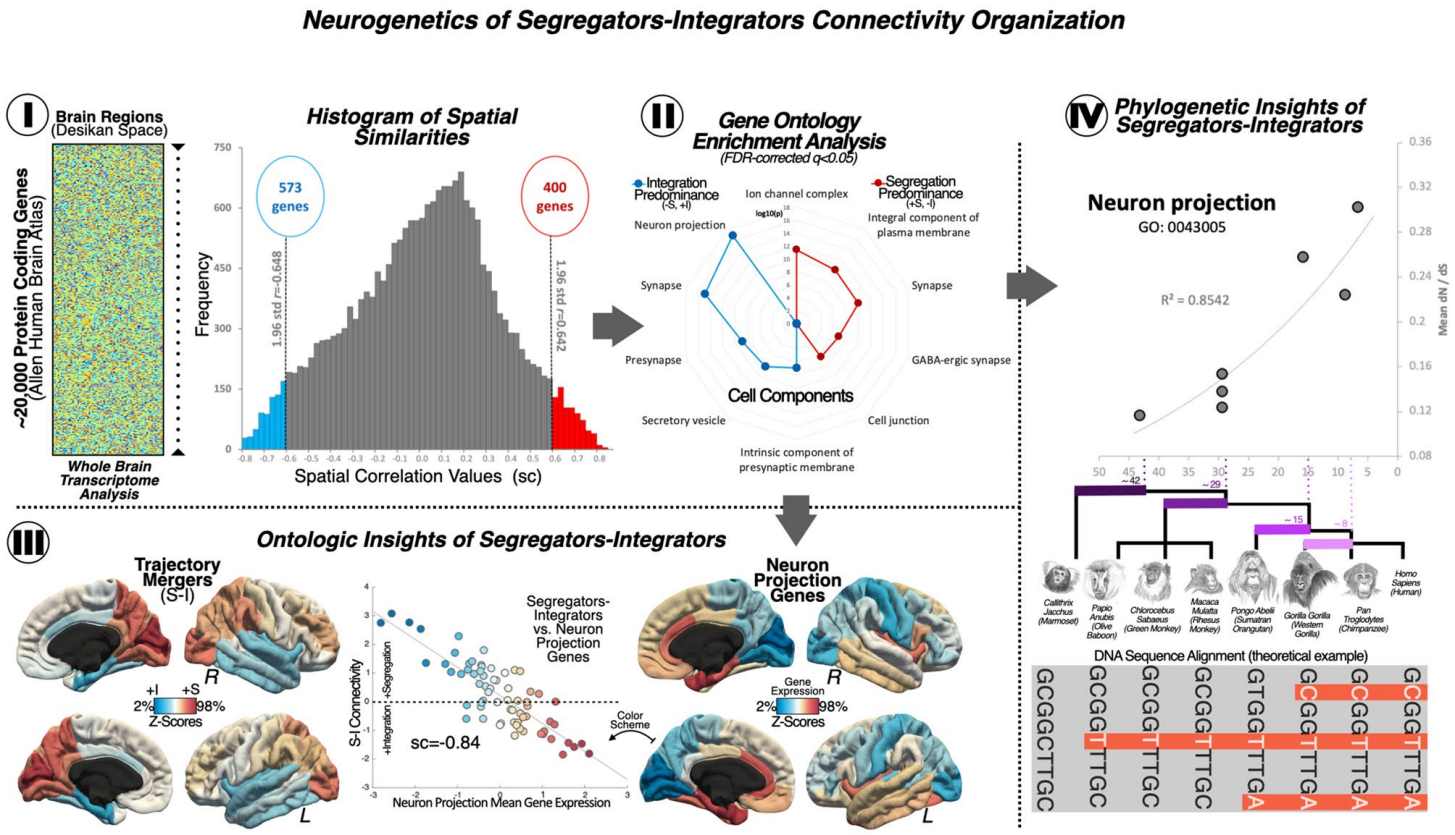
Linking brain functional organization to genetic expression. (I) Spatial similarity analysis. On the left, a representation of the whole-brain human transcriptome information from the Allen Human Brain Atlas (AHBA) distributed in the Desikan-Killiany (DK) atlas surface anatomical transformation is offered. The genetic data was organized in a bi-dimensional matrix that contained the cortical expression of 20,737 protein-coding genes from the AHBA by 68 cortical brain regions, the parcellation obtained from the DK atlas. On the right, the results of the spatial similarity are shown. Spatial comparisons were done between the mean connectivity map—resulting from subtracting the mean connectivity maps corresponding to the *early* and *late trajectory mergers*—and cortical gene expression maps from all genes of the AHBA transcriptome. A threshold set at ± 1.96 standard deviation above or below the mean was used to identify the genes with statistically significant similarity scores. In the histogram, the area highlighted in blue corresponds to the *early trajectory mergers*, while the area in red represents the *late trajectory mergers*. (II) Enrichment analyses. Using data from the Gene Ontology resource we investigated the cellular components linked to the genes related to the *early* or *late trajectory mergers*. In the radar chart, the blue dots represent the cellular components associated with brain areas showing an integrating predominance, and the red dots represent those components associated with brain areas displaying a segregating predominance. The numbers inside the radar chart are the *p* values. The results are statistically significant and corrected for multiple comparisons (FDR *q* < 0.05). (III) Ontologic Insights of Segregators-Integrators. The relationship between the trajectory mergers mean connectivity map and the brain map capturing the cortical expression of the neuron projection has been illustrated. A high correlation is found between these two maps, indicating that brain areas with integrating tendency are related to the expression of the neuron projection cellular component. In relation to the trajectory mergers mean connectivity map, brain areas in red tones are related to segregation while blue tones are related to integration. The same color scheme was used for the neuron projection brain map and the line chart, where warmer tones indicate higher gene expression. (IV) Phylogenetic insights of segregators-integrators. The line chart and the coefficient of determination (R^2^) represent the regression analysis results that explored the relationship between the most significant cellular component namely *Neuron Projection* and important evolutionary events for the *Homo Sapiens* lineage (i.e., the X axis represents divergent moments expressed in million years ago). This evolutionary event, highlighted in purple tones, reflect the emergence of separation of species from oldest (i.e., marmoset, 42.6 MYA) to newest (i.e., human, 8.8 MYA). *Note*: Color scale represents the 2–98% of the normalized connectivity data. *R* Right hemisphere, *L* Left hemisphere, *S–I* Segregating–integrating nodes; + *S* Segregation predominance, + *I* Integration predominance, *sc* Spatial correlation or spatial similarity, *std* Standard deviation, *GO* GeneOntology, *dN/dS* Biological selection ratio, *DNA* Deoxyribonucleic acid.

After obtaining the list of the cellular components associated with the genetic profiles linked to the two connectivity networks, the evolutionary aspect of these neurogenetic relationships were investigated. Using the discovered set of genes related to each connectivity profile and enrichment annotation, as well as the dN/dS ratio for each simian species, evolutionary aspects were studied between the Homo Sapiens and seven other non-human primate species (i.e., the chimpanzee, the gorilla, the orangutan, the macaque, the olive baboon, the vervet AGM, and the marmoset) (see Fig. 2-IV). Curve fitting models were used to explore the neurogenetic biological insights of the simians’ phylogeny. Bearing in mind the purpose of our study, we highlight the results regarding neuron communication functionalities, for instance: *Neuron Projection components*, we found the following curve-fitting: F = 30.99 *p* < 0.003 R^2^ = 0.86; and Synapse components, F = 30.53 *p* < 0.003 R^2^ = 0.85. All curve-fitting model results are summarized in Supplementary materials Table 7. Additionally, the robustness of these analyses was tested using a random permutation analysis approach. As described in the methods section, one-hundred iterations were calculated to obtain adjusted models for each component [adjusted determination coefficients (R^2^) with corrected *p* value]. The permutation analysis results confirmed some of the components, highlighting those related to neuron communication (*Neuron projection p* < 0.001; *Synapse p* = 0.004). The permutation analysis results are summarized in Supplementary materials Table 7.

## Discussion

The human brain orchestrates cognitive and behavioral features that differ from non-human primates but are rooted in common ancestors. In this study, we sought to uncover specific links between the functional spatiotemporal organization of the human brain, measured by means of rs-fMRI, and the evolution of the *Homo Sapiens* lineage at the biological level, using gene expression information from AHBA, gene-cell relations from the Gene Ontology resource and evolutionary divergent data from the Ensembl-BioMart online database. We developed a graph-theory analytical approach to characterize functional connectivity in a link-by-time manner, then, the cortical expression of protein-coding genes spatially related to the discovered networks was studied, as well as the cellular functionalities of these genes. Finally, we used the obtained neurogenetic evidence to explore how cellular components have persisted through years of evolution in the recent *Homo Sapiens* phylogenetic tree. Although previous research in this field has been extensively developed in relation to different aspects related to brain size or cortical expansion (e.g.,^1,3,4,6–8,47^, alternative work has implicated important neurobiological aspects in cellular, molecular and genetic features (e.g.,^8,9,48^). In this sense, investigating the specialization of the human brain is feasible from many perspectives, one being the use of resting-state functional connectivity, which is sufficiently constrained by anatomical connectivity to be a reliable source for establishing inferences of brain systems’ organization^46,48,49^. Following this rationale, our connectomics investigation (Fig. 1) yielded two robust and distinct functional connectivity maps. One related to brain segregation, observed as a fast-merging connectivity profile comprising the association fronto-parietal cortex along with the visual cortex; and a second related to brain integration located at insular and temporal regions. Minimum overlap was observed between these divergent functional maps.

The emergence of the AHBA by the Allen Brain Institute as a resource that provides whole-brain genome-wide transcriptional profiles^27,50^ has become widely known as an excellent tool for investigating the biological diversity of the human brain, at its molecular, functional and architectonic levels^51^, thus enabling connectomic-to-genomic hypothesis testing^52^. In the present study, we relate the cortical gene expression available in AHBA to our functional connectivity networks by conducting a spatial similarity analysis (Fig. 2). We identified two sets of genes whose cortical expression had highly significant spatial similarity, one set of genes was more related to the visual and association fronto-parietal areas (i.e., the early-trajectory network), while the other was more associated with the insular-temporal areas (i.e., the late-trajectory network). Using the BioMart data-mining tool within the Ensembl software environment^53^, we observed that the connectomic-genetic related gene sets exhibited overrepresented cellular functionalities important for neuronal communication. Specifically, the *Neuron projection* cellular component, which involves “any process extending from a neural cell, such as axons or dendrites” appeared as a relevant result of the enrichment analysis. This component is associated with “the process whose specific outcome is the progression of a neuron projection over time, from its formation to the mature structure”. The expression of the genes related to Neuron projection was positively related to brain areas attached to integration, while segregation would be negatively linked to this cellular functionality. Other functionalities found were as well related to neuronal processes, such as Synapse, which involves “the junction between an axon of one neuron and a dendrite of another neuron, a muscle fiber or a glial cell”, thus, is closely related to the Neuron projection cellular component, and its expression could be understood in the same sense.

In classical taxonomies, simians are an infraorder of primates which, phylogenetically speaking, belong to the *mammalia* class. The simian divergent evolutionary pathway dates back to ~ 60 million years ago and contains two main categories of species, namely, the New World monkeys, such as the marmoset, and the Old-World monkeys, like the macaque, the gorilla, the chimpanzee and the human. In relation to brain structure and function, several hypotheses have been proposed to elucidate the origin of the observable behavioral and cognitive differences between humans and their close simian ancestors (for a review see^54–56^). Some authors have centered their efforts on comparing the human brain to other simians’ brain features, for example, in cortical size^57,58^; for a review see^59^), or in number of neurons and cellular density^60^. Other authors have investigated the intersection between genetic expression and evolutionary divergences while trying to understand which specific genetic mutations have led to human cognitive phenotypes^61–65^. In our study, we are proposing another complimentary approach to previous frameworks. A combination of connectomics, genetics and divergence times, could be fruitful for better understanding what make humans unique. We used the dN/dS ratio^44^—a measure of genetic adaptation through years of evolution—and divergence times (https://www.ensembl.org/info/about/speciestree.html) as objective measures signaling moments when key genetic and epigenetic phenomena happened as each simian species evolved independently from their ancestors (e.g., the moment chimpanzees and humans separated). In general, the results found support that the *Neuron projection* functionality has been conserved through the human lineage, although, as the brain phenotype map used for the connectomics and genetics information represented both the early-mergers network and the late-mergers network, its positive expression would be related to the early-mergers network. Overall, it could be said that the functional products derived from the transcriptional expression of these protein-coding genes, which are in turn related to current spatiotemporal human brain networks, might have been positively selected to remain in the species due to their biological benefits in the brain. One possibility is that this positive selection has been key for the species to adapt and survive.

While we have been able to relate connectomics to genetic and evolutionary aspects of the human brain, we used a limited exploratory approach to provide some insight on this relationship. There are still several questions that remain unresolved and open to scientific investigation. From a neurogenetic perspective, we believe that upcoming research could be focused on the utility of in vivo neuroimaging techniques to expand our knowledge of the evolution of the human lineage. In this line, a goal would be to offer a full explanatory link between brain topological maps derived from connectomic approaches—as a means of describing cortical and subcortical functional organization—to evolutionary landmarks attached to human uniqueness, such as with the genotype–phenotype relation that gives rise to distinct cognitive and behavioral human features.

## Conclusions

The combination of the merging trajectories connectomics approach—applied to human brain in vivo data—with genetic, cellular, and evolutionary data elucidates the relationship between biological adaptive changes in the *Homo Sapiens* lineage and the functional architecture of the human brain. Implementing analytical approaches that simplify the spatiotemporal organization of human networks while conserving most of its information and respecting the network properties—i.e., summarizing the brain connectome at the link-level—makes it possible to investigate complex network relations without changing the intrinsic features as well as adding other biological variables that give more complete descriptions of the origin and current configuration of the human connectome. Our findings suggest that a balance evolved between module segregation and systems integration at the functional level. This sophisticated organizational system seems to be related to important gene-cell functions, such as those related to neurite cellular functions, that have been conserved through positive selection in the *Homo Sapiens* lineage. One of the challenges for evolutionary neuroimaging is linking valuable genetic information from *Homo Sapiens* lineage with neuroimaging evidence that simultaneously captures spatiotemporal complex properties of the human brain connectome. For instance, improving the description of the hierarchical spatial organization of the functional connectome into discrete modules at multiple levels of organization (intra and inter-modules) considering information derived from structural connectivity and, on the same time, detailing intrinsic dynamic properties within and between brain networks. Another challenge for future works is continuing the combination of biological variables with neuroimaging evidence—genetics/cellular, evolution and functional connectivity—in novel ways that could give more precise information of the individual connectome.

## Methods

### Participants

The discovery sample consisted of 80 participants (43/37 F/M; mean age = 21.60 years old, SD = 2.89; range = 19–33 years old) from The Brain Genomics Superstruct Project database (publicly available in Harvard Dataverse Repository: https://doi.org/10.7910/DVN/25833). The replication sample consisted of 80 participants (51/29 F/M; mean age = 21.68 years old, SD = 3.00; range = 19–31 years old) also from The Brain Genomics Superstruct Project database. Participants completed a full MRI and neuropsychological protocol (details available on^66^). The high-resolution anatomical scan and the resting-state scan were used in the present study.

### Data acquisition

According to^66^, images were acquired on a 3.0 Tesla Siemens TIM Trio scanner (Siemens Healthcare, Erlangen, Germany) at Harvard University and the Massachusetts General Hospital using a 12-channel phased-array head coil. The acquisitions covered the whole brain including the entire cerebellum. Slices were aligned to the AC-PC plane. Firstly, high-resolution T1-weighted multi-echo MPRAGE images were acquired as structural data (TR = 2.2 ms, TE = 1.5/3.4/5.2/7.0 ms, flip angle = 7°, 1.2 mm^3^ isotropic voxels, 144 slices). Then, functional images corresponding to the resting-state scan were acquired using a gradient-echo EPI sequence sensitive to BOLD contrast (TR/TE = 3000/30 ms, flip angle = 85°, 3 mm^3^ isotropic voxels, 124 volumes). For resting-state scans, participants were instructed to stay awake and still, with their eyes open and blinking normally.

### Image pre-processing

Preprocessing was carried out using FMRIB Software Library (FSL, version 5.0.7, https://fsl.fmrib.ox.ac.uk/fsl/fslwiki/FSL^67^), and Matlab software (version R2017a, Natick, Massachusetts: The MathWorks Inc. https://www.mathworks.com/products/matlab.html). In relation to the anatomical data, MR images were: (i) re-oriented to the anterior commissure—posterior commissure (AC-PC) plane; (ii) brain skull stripped; (iii) segmented into gray matter, white matter, and cerebrospinal fluid; (iv) normalized to the Montreal Neurological Institute brain template (*MNI152 brain template*). Then, the functional MR images were processed. The four initial data time points of the functional MRI data sets were discarded, ensuring the signal stabilization in the remaining images. The preprocessing included the following steps: (i) slice timing acquisition correction for interleaved ascending acquisitions (using the middle slice as the reference); (ii) realignment using the middle functional volume and head motion correction using a six parameter rigid body linear transformation; (iii) intensity normalization; (iv) regression of noise signals: applying a 12-parameter model (6 parameters from rigid body linear transformation and their temporal derivative) and applying the component based method CompCor for the reduction of noise (with 5 parameters from cerebrospinal fluid signal and 5 parameters from white matter signal); (v) normalization to the MNI152 brain template (3 mm^3^ isotropic); (vi) smoothing with a 6 mm full-width-at-half maximum (FWHM) isotropic Gaussian kernel; (vii) band-pass filtering retaining BOLD signal between 0.01 Hz and 0.08 Hz; (viii) data motion-censoring step (i.e., scrubbing of the time points with excessive motion) was performed through interpolation spline according to^68^, with the frame displacement (FD) threshold set to FD > 0.5 mm—no participants had excessive head motion; (ix) finally, for computational efficiency, the data were down-sampled from 3 to 6 mm^3^ voxel size.

### Image post-processing: functional matrices

In-house code was developed for the *merging trajectory analysis*, run in Matlab software. All analyses reported in this section were conducted at the individual level. Functional matrices (*r-values* and *p values* matrices) were obtained by means of calculating the Pearson’s product-moment correlation coefficients of the time series in a voxel-wise and pairwise manner (see Fig. 1-I). For doing so, a whole-brain mask of 6185 voxels covering the entire brain, including subcortical areas and the cerebellum, was applied to extract the blood-oxygen-level-dependent time series. Then, positive correlations were retained, and negative values were removed from the functional matrices to minimize ambiguity in interpretability^69^. The rate of false positives was corrected using a False Discovery Rate (FDR) approach^45^ and a correction threshold of *q* < 0.001 at the voxel level. FDR-correction was applied to the individual *p values* matrices, allowing to retain the *r-values* associated with the corrected *p values* (please see in Supplementary Fig. 1 other approaches: (i) uncorrected matrices, (ii) FDR-correction threshold of *q* < 0.05, and (iii) FDR-correction threshold of *q* < 0.0001). Finally, a Fisher’s *z*-transformation was applied to normalize the corrected correlation coefficients within the functional matrices.

### Merging trajectory analysis of the human brain connectome

In-house Matlab code was developed to investigate the spatiotemporal configuration of the human brain connectome as minimal graphs, aiming to capture its segregating and integrating properties while respecting its principles: efficiency maximization and energy cost minimization. In this sense, our graph-based *merging trajectory analysis* is intended to reduce the complexity of the functional connectome to a manageable expression that still conserves all the information of local connections and distributed large-scale networks, namely *minimal graphs* (see Fig. 1-II). The *merging trajectory analysis* was done at the individual level using a whole-brain voxel-wise analytical approach. The FDR-corrected functional matrices from the prior step were used as input in this analysis. To reduce dimensionality of the network, we selectively merge pairs of nodes which share high connectivity patterns. To determine whether or not to merge two nodes, we compute the weighted degree of all common and distinct links between these two of nodes. If the weighted degree of the shared links is greater than the weighted degree of the distinct links, then we merge the two nodes. If the weighted degree of distinct links is higher than that of the shared links, then we proceed to the next set of nodes. This process is performed iteratively, beginning with the strongest connections in the network and moving in descending order through the network, until we have obtained the minimal graph. The connectivity of the merged node is computed as the mean strength for each link. Following this method, the rest of the nodes are successively organized and included to this network. In Supplementary Fig. 3 two different moments (one earlier and one later) of the merging process have been represented from a graph perspective to illustrate how the merging trajectory is computed. Additionally, we tested whether using our merging rule against using a random merging rule generated different connectivity results. In Supplementary Fig. 4 a comparison of how cumulative number of segregated and integrated links vary during the merging process can be found, where the logarithmic of the number of links has been plotted for the rule described in here *vs.* a random rule. It is possible to observe that a random rule does not perform well the merging process, and using the rule described in here a high difference in the amount of segregated and integrated links merged through time, indicating that the rule follows a pattern rather than selecting indistinctively segregated or integrated links. Once the *merging trajectory analysis* is applied to each participant’s data, the next step consisted in estimating the best curve-fitting model to the data. Two different regression models were applied: logarithmic fit and exponential fit. Next, the data was normalized at the individual level, and mean group-level matrices for each regression model were obtained. The CARET software (http://brainvis.wustl.edu/wiki/index.php/Caret:About) was used for projecting the network results on to brain volume (see Fig. 1-III).

### Neuroimaging, genes and evolution of the central nervous system

To study the relation between connectomics and genetics, we followed the approach described in previous research (e.g.,^16,25,29,43^), however, we have updated our pipeline according to a recent publication^70^. We used the Allen Human Brain Atlas (AHBA)^42^ to investigate the spatial similarities between protein-coding genetic profiles and the functional connectivity profiles obtained in the previous step. For this analysis we used the mean FC map, which was obtained by subtracting the early-mergers network map *minus* the late-mergers network map. The AHBA provides whole-brain genome-wide expression patterns for six human subjects^27^. For the spatial similarity strategy, we used the surface anatomical transformation of the transcription profiles, which includes 20,737 protein-coding genes, based on 58,692 measurements of gene expression in 3702 brain samples obtained from those 6 adult human subjects^71^. The surface anatomical transformation is based on the 68 cortical regions of the Desikan-Killiany atlas^72^, which covers the entire cortex and uses individual vectors of the median cortical expression of the genes across the 68 cortical regions. Specifically, three processing stages were followed to derive the Desikan projected transcriptome data: (i) expression values from multiple probes were mean averaged for each gene; (ii) each sample was mapped to a cortical region of the Freesurfer Desikan atlas. All samples with an anatomical annotation (provided by Allen Institute) located outside the cortex were removed. Samples within the Desikan atlas cortical regions were automatically mapped and an extensive review of the Allen samples outside these voxels was made (56% of all cortical samples). The closest Desikan cortical region and the Allen reference atlas annotation were used to weight the mapping decisions; and, (iii) the average genetic expression across all samples was mapped into a specific Desikan atlas region, and this process was computed for each individual brain. A group expression map was derived computing the median values between the 6 donors. The specific steps of our connectomics-genetics pipeline are as follow: (*step I*) the brain phenotype map—mean connectivity map obtained by subtracting the early-mergers network map *minus* the late-mergers network map—was spatially correlated with ~ 20,000 genes from the AHBA. The obtained correlation values, represented by $\pm$ 1.96 standard deviation, were stored. (*step II*) 1000 surrogate maps with spatial autocorrelation matching the original image were generated using BrainSMASH (Burt et al. 2020 numbered citation). For each of the surrogate maps, two additional computational steps were completed: (*a*) a correlation with the ~ 20,000 genes included in the AHBA, saving all the genes with a correlation higher than the cut-off value obtained in the first point. We used this threshold instead of computing the threshold on the new distribution to avoid obtaining correlation values arising due to spatial autocorrelation; and (*b*) an overrepresentation analysis of the obtained list of genes using a Fisher test, where a *p* value and fold enrichment were computed for each annotation. (*step III*) Overrepresentation of the upper and lower bounds of the original distribution were computed: for each annotation, a Fisher test was applied, and the obtained *p* value was compared with the *p* values obtained for the surrogate maps in the same annotation. A *p* value was computed indicating how likely that annotation can appear in the surrogate maps. False discovery rate was used to correct this *p* values for multiple comparisons. The *p* values that did not survive the multiple comparisons test were discarded. (*step IV*) To help the interpretation of the results, the resulting annotations were grouped into clusters based on a similarity approach^73^. The following steps were done for clustering the annotations: (*a*) a binary gene-term matrix was generated. Genes belonging to a particular term had a value of 1 and 0 otherwise; (*b*) a term-term kappa score matrix was generated. Each entry measured the similarity of 2 terms with kappa score based on observed occurrence of genes and was compared to chance occurrence; (c) hierarchical clustering was applied were terms with a kappa threshold > 0.3 were merged; (*d*) for each cluster the term with the most significant *p value* was used as the representative term. Finally, (*step V*) the enrichment analysis has been carried out for *Cellular Components* terms from Gene Ontology (the reference genome and Gene Ontology annotations were retrieved from http://www.webgestalt.org/ on 01/14/2019). Additional analysis details were as follow: (*a*) genes that were not annotated as *Cellular Components* were not used for the analyses; (*b*) terms with less than 5 or more than 2000 genes were removed; (*c*) annotations with less than 3 genes overlapping with the candidate gene list or with less than an uncorrected *p *value of 0.01 (Fisher test) were discarded. The spatial similarity analysis was done by means of in-house Matlab code. We built a null hypothesis distribution by comparing the entire transcriptome with the mean subtraction map, and applied cut-off thresholds to identify the most relevant genes. To test that the correlations were not due to spatial autocorrelated properties we generated 1,000 surrogate maps with spatial autocorrelation matching to original image using BrainSMASH software^74^. To generate SA-preserving surrogate brain maps, BrainSMASH produces random maps whose variograms are approximately matched to a target brain map’s variogram. A distribution with 20,736,000 correlations was generated (1000 random maps × 20,736 genes) to test the correlations threshold for a statistically significant correlation *p* value < 0.05. In the positive tail of the distribution, correlations higher than 0.4672 are defined as significant and not due to spatial autocorrelation properties. Similarly, in the negative tail, a correlation of -0.4690 would be significant. For the subsequent overrepresentation analysis, we took genes with correlation higher than *r* = 0.6421 and lower than *r* =  − 0.6481 ($\pm$ 1.96 standard deviation from the original correlation distribution), where all of them are statistically significant based on this permutation analysis (*p* value < 0.0014) (see Supplementary materials, Tables 1–6).

The Ensembl resource (https://www.ensembl.org/;^53^) was used to compare different species from the Homo Sapiens lineage to the Homo Sapiens itself (*i.e.,* homologue-orthologue comparison). The seven species compared were: the chimpanzee (*Pan Troglodytes*), the gorilla (*Gorilla Gorilla*), the orangutan (*Pongo*), the macaque (*Macaca*), the olive baboon (*Papio Anubis*), the vervet AGM (*Chlorocebus pygerythrus*), and the marmoset (*Callinthinx Jacchus*). The selection of the species was based on phylogenic divergence time (in million years: 6.6 8.8, 15.8, 29.4, 29.4, 29.4, 43.2, respectively; https://www.ensembl.org/info/about/speciestree.html), which indicates important biological landmarks such as changes in genomic configuration. The specific data extracted from the Ensembl resource were the dN value and the dS value, that we used to calculate the dN/dS ratio. This measure has been indicated to be useful for assessing the strength of natural selection acting on protein-coding genes^75^. In evolutionary biology studies, dN/dS ratio has been widely used because it has a straightforward interpretation: the excess rate of amino acid-replacing non-synonymous substitution compared to silent synonymous substitution (ω > 1) indicates positive, adaptive, or diversifying selection, while the reverse direction (ω < 1) indicates negative or purifying selection^76^. In this sense, it is an informative measure of the evolutionary rates of protein-coding genes, or in other words, biological features that have been conserved in the species during evolutionary adaptive periods^75^. The main reason for using the dN/dS ratio is its ability to capture non-synonymous mutations. While synonymous mutations are mostly considered as having neutral effects on the organisms (i.e., do not change the sequence of amino acids of protein coding genes), non-synonymous mutations can be positive (advantageous) or negative (deleterious). This distinction is important because neutral mutations occur at the same rate as genomic mutations (μN), but positive and negative mutations are affected by natural selection (μS), occur at faster or slower rate respectively, and are different than neutral mutations (μN ≠ μS). When calculating the rate synonymous: non-synonymous we are calculating the mode of natural selection, introducing the time of divergence (in the sequence alignment of the orthologues genes within a phylogeny) in the equation allows to measure the strength of the mutation (dN = tμN and dS = tμN), thus leading to ω = dN/dS (see^75^). When performing this analysis, the following parameters were selected in BioMart software^53^: (i) the human gene GRCh38.p13 as dataset; (ii) the set of genes specifically related to each cellular component annotation as the external input in the filter section; (iii) and as attributes for the comparison the homologues option and the orthologues (dN, dS) for each species. To automatize this process, we used R software^77^ was used to retrieve the dN and the dS values from a public database. The obtained data were saved to a database, where the dN/dS ratio was calculated for each annotation and each species homologue-orthologue comparison (dividing the dN value by dS value), and the averaged dN/dS ratio was used for subsequent regression analysis. Using IBM SPSS Statistics software (version 28 Armonk, New York, USA), regression models were calculated for studying the relationship between the mean dN/dS ratio and the years of evolution of the species. This analysis was done using a curve estimation module within the regression analysis tool of the SPSS Statistics, where the years of evolution were entered as independent variable and the mean dN/dS ratio of each homologue-orthologue comparison, and each cellular component annotation was introduced as dependent variable. Regression models were done separately for the upper or lower bound of the null hypothesis distribution. Four different fitting models were tested: linear, logarithmic, quadratic and exponential; the best curve fitting amongst these four regression models was selected in terms of R^2^, F-statistic and *p *value.

In order to test the robustness of the regression analysis, a random permutation analysis approach was used to build null distributions and calculate a corrected *p* value per regression model. The following steps were followed for each original regression model that surpassed the multiple-comparisons threshold correction (*p value* < *0.*05): (i) generating 100 sample lists with *N* genes in each new list (*N* as the total number of genes from the original component list) by means of in-house code using Matlab that followed these steps: first, loading the original list and excluding from the analysis the genes that were already present in the original component list; second, randomly selecting *N* genes from the 20,737 protein-coding genes from the AHBA^42^. In this step we used the Matlab *randperm function* (https://www.mathworks.com/help/matlab/ref/randperm.html); (ii) repeating the homologue-orthologue comparison between humans and the other seven *Homo Sapiens* species like in the original analysis: obtaining for each new list the dN score and dS score using R software to access the Ensembl-BioMart environment and computing the mean dN/dS ratio for each species; (iii) running the curve fitting estimation analysis in R-software and IBM SPSS Statistics (version 28) using the mean dN/dS ratios for the seven non-human primates and the evolutionary divergence times as input data, and obtaining R^2^, F-static and *p *value for each iteration. In this step the selected model for the new list matched the best curve fitting model for the original analysis (e.g., if the original list of the *Neuron projection* component was best fitted with an exponential function, then the same regression model was used for fitting the data in the new one hundred samples). From this analysis the normalized determination coefficients (null distribution) were saved for the next step; (iv) calculating the corrected *p* value for the original list: first, calculating the mean and the standard deviation of the null distribution (normalized determination coefficients); second, obtaining the *z*-score for the original *p* value (i.e., using the mean and the standard deviation from the previous step); third, obtaining the corrected *p* value by calculating the cumulative distribution function using the Matlab Mathworks *normcdf function*.

## Supplementary Information

Supplementary Information.

## Data Availability

The functional MRI data and the genetic data that supports the findings of this study are available from the Brain Genomics Superstruct Project GSP (http://neuroinformatics.harvard.edu/gsp), the Allen Human Brain Atlas project (https://portal.brain-map.org).

## Code availability

All codes for imaging analysis are available for the research community from the corresponding author (J.S.) upon reasonable request.
