# Supplementary material for: Divergent connectomic organization delineates genetic evolutionary traits in the human brain
Source: Sci Rep. 2021 Oct 4;11:19692. doi: 10.1038/s41598-021-99082-6 (PMC8490416; doi:10.1038/s41598-021-99082-6)

Supporting Information

Supporting information document for "*Divergent Connectomic Organization Delineates Genetic-Evolutionary Traits in the Human Brain*" by Elisenda Bueichekú, Jose M. Gonzalez-de-Echavarri, Laura Ortiz-Teran, Victor Montal, Federico d'Oleire Uquillas, Lola De Marcos, William Orwig, Chanmie Kim, Elena Ortiz-Teran, Silvia Bassaia, Ibai Diez, Jorge Sepulcre.

Corresponding author

Correspondence should be addressed to: Jorge Sepulcre (sepulcre@nmr.mgh.harvard.edu).

Index

The document contains the following sections:

1. **Supplementary tables**

- Supplementary table 1. Genes linked to the upper bound of the probability distribution.
- Supplementary table 2. Genes linked to the lower bound of the probability distribution.
- Supplementary table 3. Cellular components linked to the upper bound of the probability distribution.
- Supplementary table 4. Cellular components linked to the lower bound of the probability distribution
- Supplementary table 5. Specific genes related to the cellular components linked to the upper bound of the probability distribution
- Supplementary table 6. Specific genes related to the cellular components linked to the lower bound of the probability distribution
- Supplementary table 7. Original curve fitting model analysis results and permutation analysis results

2. **Supplementary figures**

- Supplementary Figure 1. Validation of the main connectomics results
- Supplementary Figure 2. Replication of the main connectomics results
- Supplementary Figure 3. Illustration of the merging process
- Supplementary Figure 4. Comparison of the merging process to a random assignation process

1. SUPPLEMENTARY TABLES

Supplementary table 1. Genes linked to the upper bound of the probability distribution.

SCN1A	LOC254559	VSTM2A	STX8	KCNT1
SCN1B	ATP6V0A2	PRDM5	TRIM7	KCNS1
GALR1	FOXO3	PRDM2	CACNB4	ENTPD4
ST8SIA1	STAMBPL1	SLC24A2	NUAK1	THAP10
NR2C1	FOXP1	NPM2	ARHGEF18	MSL2
SCN8A	C11orf52	OXCT2	CACNG2	SETD7
DCLK1	CCNB1IP1	LOC100134173	JDP2	RORB
TTC21B	EXTL2	CMYA5	PASK	RORA
ATXN1	TRPM3	ZNF684	ANKRD34C	CDR2L
OR2L3	TRPC3	ZNF35	LOC100288939	DMKN
LYSMD4	SNRPN	ZNF20	MIR31HG	NFKBIZ
NR3C2	STAG3L4	RET	JARID2	FAM71E1
NR3C1	TAF4B	RAD54B	LRRN3	MYO19
OPN3	GRIN2A	CDKL1	MSRB3	NKX3-1
MAFB	SRSF1	C10orf140	FBLN7	C6orf47
TTC39C	P2RX6	KIAA1797	SLC25A37	STS
TTC39B	A26C1B	GPLD1	SSX2IP	GRHL2
NFIC	SOHLH1	RP11-422J8.1	SLC25A12	ADAM22
INA	C12orf24	IL6R	NIPAL2	INPP5A
MADCAM1	KIAA0802	ZNF333	RSRC1	SHD
GK	HECW1	FMN1	RUSC1	CEP152
KCNAB3	PARM1-AS1	ZNF358	GRAMD1B	ASB13
HIST1H1D	TMPPE	GK3P	NAGPA	CRABP1
ARL4C	NAP1L2	RNF148	LNX2	SRL
CS	SERTAD4	RNF168	LOC100131943	C8orf47
IDE	PCDHB15	RNF157	AP000926.2	TNFRSF25
HR	PCDHB10	NXPH3	STXBP5L	POU3F2
CFAP221	DDX3Y	SLC45A4	FASTKD1	ICA1
PRH2	POSTN	ERMP1	FNDC5	SLC29A1
MOGAT1	C14orf104	STX19	ITPR1	FAM131B
EFHA2	PLCB4	C6orf186	SATB1	TEAD4
SORL1	PLCB1	CCNI	SATB2	DENND5B
ZADH2	ZNF792	VWC2	SLITRK3	C17orf75
PLA2R1	PAIP1	C6orf138	DSCC1	IFNE
SLC5A6	ESRRG	SPAG6	PPIEL	C9orf72
IGFBP2	GLCCI1	RHBDL3	SPAG16	C9orf45
LY6G5B	EIF4A2	CDH7	PPP3CC	ZBTB16
PLEKHH3	CDKN1B	FLT3	ACSL3	VIPR2
TCHP	C4orf18	CDS1	PVALB	CCDC39
ST3GAL6	PLAGL1	FGF18	VAMP1	CCDC58
CORO6	ZMAT4	FGF14	PPIL5	TMEM86B
AFTPH	MCF2L	MRS2	LOC100286909	RP11-403C10.2
SLC6A17	ATP4A	EGR1	ASPSCR1	CCDC136
TC2N	INTS4L1	PDSS1	KLF9	SPTSSB
TBR1	INTS4L2	EMILIN3	AGPAT9	ZNF385D
OSBPL6	MRPS30	PRAG1	MTBP	ZNF385B
RBM20	KIAA0240	ATP2B2	CADPS2	ZNF385A
ABCC8	C18orf25	DYNC2H1	ZYX	UCHL5
AADACL1	EIF5A2	RIMS3	ZDHHC5	LYPD5
CD274	NCALD	ATP2A2	KCNC1	MRPL33
ATG16L1	ECM1	RAB37	KCNC3	STARD5
ATP8B1	SETBP1	ZNF193	RASGRF1	SMC1A
GAS2	TSHZ1	GLRB	KCNA2	IL1RAP
AS3MT	STRBP	STRC	KCNA1	PLXDC1
USP45	TTLL11	FSTL1	TTBK2	PKD1
GPR89B	KIAA1107	ZNF225	KCNB1	GPR161
MIAT	BACH1	ZNF295	HIVEP2	LGI3
HS3ST1	FGF5	RG9MTD2	CHGA	LGI2
CNNM1	FGF9	PCSK1	JAM2	POU6F1
LRRC37A4P	FHDC1	ZNF268	ENPP5	POU6F2
SEMA7A	C20orf20	HAPLN4	EDNRA	CPLX1
SEMA6D	OSBPL1A	ELAVL4	AC013402.2	PKNOX2
MAP3K13	GABRD	KIAA1456	ELL2	HCN1

PIM1	ETV6	TAS2R30	RFX5	LUZP1
BMP4	C20orf100	ANKRD29	GABRG2	PPARGC1A
PION	LMTK2	SGCG	GABRG3	SLC39A14
PDIK1L	PPP1R28	YPEL4	CDC42EP3	CUX1
A2BP1	INTS9	RTKN2	IQSEC1	C1RL
PIF1	INTS8	LINC00515	KLF12	C1orf71
DCBLD2	PDIA5	ARC	PENK	C19orf46
BTBD11	RASSF5	PCP4L1	LEPROTL1	MAGI3
SERPINB9	RBAK	LRRC38	LAG3	C1orf97
PHYH	ECEL1P2	LRRC49	NCK2	TRIP11
SCRT1	ANK1	LRRC8C	SIX4	CABP1
RPH3A	ANKH	ZCCHC8	EPB41	C1orf172
TUBE1	LOC643669	TIFA	DPY19L2P4	CRHR2
TUBD1	TAS2R13	C1orf201	IL28RA	STAT4
C7orf63	ALDH1A3	GABRB2	LEPREL2	RP5-1022P6.1
UPP1	TAS2R43	TRIM37	STAC2	DNAJC5G
LINC00473	TAS2R31	GABRA1	TPTE2P6	CPNE9

Supplementary table 2. Genes linked to the lower bound of the probability distribution.

	APOE	NNAT	CXCL1	LPHN3
GDA	PLD6	ATOX1	PYCRL	CACNG3
SCN3B	LOC728392	SNX26	FLJ38379	ARMCX2
TMEFF2	CAMKV	HIF3A	CD24	ARMCX1
RALGPS2	C11orf66	FAM65B	C6orf118	GLUD2
GHR	PLB1	C4orf32	FGFR3	PGM2L1
SPATA18	FBXO2	MMD2	CD83	CCBE1
SCN9A	SEMA3D	SLC26A4	DYDC2	LOC100288911
PHPT1	GOLM1	ATOH7	SAP30BP	FAM113B
CAMK2D	SEMA4A	ZNF831	AC044839.2	CTXN1
PTPRF	ZWILCH	ARHGAP18	LSM3	TMEM176B
NUTF2	NIT2	TP53I3	TMSB4X	TMEM176A
HN1	LINC00086	GNG2	CXXC11	SLC25A23
GNG10	C1S	GNG4	KIAA1644	SLC25A22
DACH2	UBL5	RILPL2	TMSB10	EMP3
TAF7L	CXorf57	C9orf9	MRAS	C16orf55
RTBDN	DAP	SH3RF1	PYDC1	KIRREL2
KLHL1	NTSR1	GPR88	CDH4	SULF2
SLC4A3	NTSR2	GPR98	CDH9	ITPRIPL2
MAFG	CLEC4G	GNB4	SLIT1	DDAH2
ECHDC3	CFD	KIAA1244	SLIT3	UG0898H09
LOC392145	AKAP14	NCAM2	TNFRSF10B	AP3S1
PTPRA	AC006273.1	NKAIN2	LYZL4	CES7
NFIB	NUPR1	NKAIN4	LOR	LARGE
SLC30A10	CALM3	PRDX4	RIPK2	RP4-725G10.1
PEA15	ANKRD6	CAPSL	RP11-566K11.2	VAV1
OPRK1	CALB2	MDGA1	ISOC1	RSPO4
EXOC6	LOC100292909	SUSD1	SPARC	RSPO3
NTNG2	ARHGAP6	SLC27A5	AC005152.2	SH3BGRL3
TXLNB	MAPK1	PYGL	TMEM130	SNCG
S100Z	MAPK3	PTHLH	CHCHD6	SNCA
KL	PMVK	GMFB	LOC441052	NUDT10
C21orf62	AL161668.2	ANXA11	CACHD1	NUDT11
EFHC2	SLC1A4	SSTR2	TMEM108	FAM122C
STYK1	PTCHD1	SSTR1	CDH10	GYPE
C21orf67	GTF3C6	CENPVL1	FABP1	SUMO3
TG	C12orf73	FGGY	FABP7	ENO1
BIRC3	HES5	PNPLA4	FABP5	C6orf222
TMTC1	C12orf45	MOBKL1A	PTGER3	C6orf225
ARL10	C12orf53	HRH1	PTGER4	CCDC42B
PLXNC1	C12orf23	TMEM200A	ATP2B4	ST6GALNAC5
RPS27AP11	DACT1	KIF21B	MMD	PGAP1
DTNBP1	LY6H	OXCT1	CAP1	NHLRC1
IGFBP5	PNCK	HIST1H2BK	MIF	LOC145837
SLC2A4RG	CORO1A	RPS10	LOC644172	WNT7B
CDCA7	SCGN	GPD2	CASK	PRSS23
WNT10B	LINC00260	ENOX2	FNBP1L	GRAMD2
GGCT	HTR1A	GPC4	SEPW1	KLK7
SMARCD3	WDR86	TTPAL	AP000654.2	RSPH9
PSD3	LINC00282	RASAL1	SVOP	KLF8
CDC42	PCDH15	C10orf116	TMEM158	HPCAL4
RASL10A	PCDH17	FAM70A	TMEM159	VBP1
RASL11B	WDR66	RAB27B	MAPK8IP1	FKBP1A
B9D1	WDR69	JUN	MYB	FKBP1B
PSMB2	HTR3A	C17orf108	PCSK5	HRASLS
CD244	HTR2C	DUSP9	CMTM8	SNX7
MGST1	C13orf36	AC093310.1	CBFB	RRAGB
NECAB2	LOC643037	RNASEH2A	CCDC90B	TSKU
TXNL4A	GTF2F2	RNASEH2C	MYL12B	PRKCG
PKIB	PNMT	IL33	TRIM6	PRKCD
PKIA	LOXL1	IL34	NOV	RP11-60I3.5
APLN	F12	RNF150	OBFC2A	UBE2S
TBCA	UHMK1	PTPLAD2	SUB1	C3orf78
FIBIN	PON3	CD63	RABL5	KCNJ1
MESP1	PLCB2	C6orf173	LOC653051	AP1S1

KCNG1
C3orf26
MT1A
MT1L
MT2A
DLL3
TUBB
ORC6L
ALDH9A1
C2orf80
ZCCHC12
ZCCHC18
ZCCHC17
GNPTAB
LRP1B
ELL3
TIMM8B
SOX5
RP11-544M22.1
C3orf14
LASS1
KCNQ2
SYNPR
FAM107A
SLC17A8
KCNN3
FAM150B
TMEM120A
ELMO2
TXN
SNAP29
FAM71F1
ASCL2
IL12RB2
DMC1
CLSPN
GRIA1
C6orf52
NT5DC3
ROBLD3
KCTD4
TPO
FILIP1
PPP4R4
ETNK2
TUBB2A
DAPL1
SYTL5
GLRA2
SQLE
EFEMP2
MAN1A1
PDLIM5
RAVER2
RAPGEF4
LYRM2
DMWD
WBP1
DMRTC1B
LIX1
GPRIN2
C8orf46
TBC1D26
GUK1
ST5
SLA
SP5
LIPH
MYH7
TOX3
C17orf89
IL13RA2
DOK6
CMAS
TMEM106C
GLOD4
SYT17
GRM1
GLP2R
DISC1
DYNLL1
AC093283.3
UCHL1
CCDC103
CCDC112
MRPL49
LOC339524
TNNT1
EFCAB1
FAM149A
FAM148C
SPRR2G
TPM3
RGS14
RGS22
RGS20
DOC2A
DUSP18
DUSP13
IGFN1
AK3L1
DPYD
COCH
MILR1
HBG1
HS2ST1
RBP4
PELI1
GPR160
ZFP64
COG1
PDGFB
NPTXR
CPLX3
DNAH5
AGXT2L1
RDBP
PJA1
CCDC109B
VAT1L
STK32B
GNAO1
EFNB2
HBQ1
PID1
CNR1
LOC100287347
CNN3
LDHD
C7orf31
RAP2B
LOC100287146
DSEL
UPP2
FAM171B
ICAM5
LINC00461
MBOAT2
ROBO1
ROBO2
C20orf112
PHEX
AC078937.4
PPP1R1A
LOC100287080
TLL1
LOC648771
BATF3
C1QL1
C1QL2
TNFAIP2
ANO3
LCP1
C12ORF75
DCAKD
C6orf1
YPEL1
DTNB
ANKRD43
ANKRD50
MOXD1
AMZ1
LOC100289263
NDUFC2
PGA3
LRRC36
LRRC42
LRRC3B
LRRC56
LRRC57
RGMB
C1orf226
WDFY4
CADM1
HSBP1
TMSB15A
RPL39L
AC010980.2
SFT2D2
GABRB1
TRIM36
PACRG
GABRA2
GABRA3
GABRA5
PDCD6
EPCAM
SEL1L2
IFI27L2
GUCY1A3
CREM
PGRMC1
POLE4
DNAJC25-GNG10
LOC100131504
RHOC
PDYN
PRODH2
SNRNP27
RPL27A
MAGED4B
ODZ3
C5orf33
EXD1
LOC642852
LOC100131482
CHID1
PTRH1
DNAJA4
LUZP2
FANCL
LOC100129309
MSANTD1
LRRC16B
C1orf50
C1orf53
C19orf12
C1orf95
LOC391722
RNASET2
PDE1A
PDE2A
SLC7A4
TIMP4
TIMP2
TIMP1
DYRK3
DYRK2
DCAF15
AC024575.2
C1orf187
CABP7
ANXA8
DPYSL3
GDPD2
SLC2A12
GCNT1
DNAJC12
LOC100129291
FZD1
FZD2
SAMD3
FZD7
THRA
CPNE6
CPNE7
LOC100131283
KCTD17
KCTD12

Supplementary table 3. Cellular components linked to the upper bound of the probability distribution.

Cluster 01: Ion channel complex

	Gene Ontology (GO)	Log10 (p-value)	Fold Enrichment (FE)	Cellular Component Annotation name
1	0034702	11.39	5.55	Ion channel complex
2	1902495	10.66	5.14	Transmembrane transporter complex
3	1990351	10.43	5.02	Transporter complex
4	0034703	8.24	5.48	Cation channel complex
5	0098796	5.73	2.25	Membrane protein complex
6	0034705	4.78	6.12	Potassium channel complex
7	0098797	4.42	2.63	Plasma membrane protein complex
8	0008076	4.24	5.99	Voltage-gated potassium channel complex

Cluster 02: Integral component of plasma membrane

	Gene Ontology (GO)	Log10 (p-value)	Fold Enrichment (FE)	Cellular Component Annotation name
1	0005887	10.21	2.46	Integral component of plasma membrane
2	0031226	9.38	2.35	Intrinsic component of plasma membrane

Cluster 03: Synapse

	Gene Ontology (GO)	Log10 (p-value)	Fold Enrichment (FE)	Cellular Component Annotation name
1	0045202	10.02	2.77	Synapse
2	0098794	8.01	3.31	Postsynapse
3	0097060	7.45	3.72	Synaptic membrane
4	0045211	7.06	4.15	Postsynaptic membrane
5	0098793	4.36	2.76	Presynapse
6	0099572	4.23	3.14	Postsynaptic specialization
7	0098978	4.08	3.05	Glutamatergic synapse
8	0098590	4.03	1.99	Plasma membrane region
9	0098984	3.68	2.93	Neuron to neuron synapse
10	0014069	3.5	2.95	Postsynaptic density
11	0032279	3.45	2.92	Asymmetric synapse

Cluster 04: GABA-ergic synapse

	Gene Ontology (GO)	Log10 (p-value)	Fold Enrichment (FE)	Cellular Component Annotation name
1	0098982	6.77	8.89	GABA-ergic synapse

Cluster 05: Cell junction

	Gene Ontology (GO)	Log10 (p-value)	Fold Enrichment (FE)	Cellular Component Annotation name
1	0030054	6.36	2.26	Cell junction

Cluster 06: Neuron projection

	Gene Ontology (GO)	Log10 (p-value)	Fold Enrichment (FE)	Cellular Component Annotation name
1	0043005	6.21	2.23	Neuron projection
2	0030425	4.79	2.63	Dendrite
3	0097447	4.76	2.62	Dendritic tree
4	0036477	4.28	2.26	Somatodendritic compartment
5	0030424	3.43	2.35	Axon
6	0043025	3.38	2.47	Neuronal cell body
7	0044297	2.71	2.18	Cell body

Cluster 07: Integral component of synaptic membrane

	Gene Ontology (GO)	Log10 (p-value)	Fold Enrichment (FE)	Cellular Component Annotation name
1	0099699	5.43	5.19	Integral component of synaptic membrane
2	0099240	5.1	4.82	Intrinsic component of synaptic membrane
3	0099055	4.97	5.7	Integral component of postsynaptic membrane
4	0098936	4.81	5.46	Intrinsic component of postsynaptic membrane
5	0099634	4.64	5.88	Postsynaptic specialization membrane
6	0099060	3.92	6.3	Integral component of postsynaptic specialization membrane
7	0098948	3.81	6.06	Intrinsic component of postsynaptic specialization membrane
8	0098839	3.04	5.33	Postsynaptic density membrane
9	0042734	2.52	3.31	Presynaptic membrane
10	0099061	2.15	5.23	Integral component of postsynaptic density membrane
11	0099146	2.06	4.94	Intrinsic component of postsynaptic density membrane

Cluster 08: GABA-A receptor complex

	Gene Ontology (GO)	Log10 (p-value)	Fold Enrichment (FE)	Cellular Component Annotation name
1	1902711	5.14	17.54	GABA-A receptor complex
2	1902710	5.03	16.66	GABA receptor complex
3	0034707	4.06	8.16	Chloride channel complex

Cluster 09: Main axon

	Gene Ontology (GO)	Log10 (p-value)	Fold Enrichment (FE)	Cellular Component Annotation name
1	0044304	5.06	7.73	Main axon
2	0033268	2.88	13.33	Node of Ranvier
3	0043194	2.5	10	Axon initial segment

Cluster 10: Neuron projection membrane

	Gene Ontology (GO)	Log10 (p-value)	Fold Enrichment (FE)	Cellular Component Annotation name
1	0032589	4.66	8.19	Neuron projection membrane
2	0032590	4.57	10	Dendrite membrane
3	0032809	3.18	9.87	Neuronal cell body membrane
4	0044298	3.18	9.87	Cell body membrane
5	0031256	2.39	3.16	Leading edge membrane
6	0031253	2.31	2.39	Cell projection membrane

Supplementary table 4. Cellular components linked to the lower bound of the probability distribution.

Cluster 01: Neuron projection

	Gene Ontology (GO)	Log10 (p-value)	Fold Enrichment (FE)	Cellular Component Annotation name
1	0043005	16.90	3.93	Neuron projection
2	0030424	9.94	4.37	Axon
3	0036477	9.81	3.79	Somatodendritic compartment
4	0030425	8.57	4.06	Dendrite
5	0097447	8.53	4.04	Dendritic tree
6	0044297	6.49	3.75	Cell body
7	0043025	5.13	3.57	Neuronal cell body
8	0150034	3.69	3.81	Distal axon
9	0044306	2.67	4.32	Neuron projection terminus
10	0043679	2.48	4.41	Axon terminus
11	0030426	2.20	3.66	Growth cone
12	0030427	2.16	3.61	Site of polarized growth

Cluster 02: synapse

	Gene Ontology (GO)	Log10 (p-value)	Fold Enrichment (FE)	Cellular Component Annotation name
1	0045202	14.46	3.87	Synapse
2	0097060	9.59	4.96	Synaptic membrane
3	0098590	6.59	2.89	Plasma membrane region
4	0045211	6.14	4.57	Postsynaptic membrane
5	0030054	5.91	2.73	Cell junction
6	0098794	5.01	3.31	Postsynapse
7	0098978	4.47	3.81	Glutamatergic synapse
8	0099572	3.21	3.33	Postsynaptic specialization
9	0098984	2.59	3.13	Neuron to neuron synapse
10	0005911	2.29	2.72	Cell-cell junction
11	0014069	2.17	2.95	Postsynaptic density
12	0032279	2.14	2.92	Asymmetric synapse

Cluster 03: Presynapse

	Gene Ontology (GO)	Log10 (p-value)	Fold Enrichment (FE)	Cellular Component Annotation name
1	0098793	8.92	4.55	Presynapse
2	0030658	4.88	5.05	Transport vesicle membrane
3	0030133	4.56	3.74	Transport vesicle
4	0030672	4.37	6.53	Synaptic vesicle membrane
5	0099501	4.37	6.53	Exocytic vesicle membrane
6	0070382	4.29	4.74	Exocytic vesicle
7	0098563	3.50	8.69	Intrinsic component of synaptic vesicle membrane
8	0008021	2.94	4.07	Synaptic vesicle
9	0030285	2.37	8.08	Integral component of synaptic vesicle membrane

Cluster 04: Secretory vesicle

	Gene Ontology (GO)	Log10 (p-value)	Fold Enrichment (FE)	Cellular Component Annotation name
1	0099503	8.28	3.35	Secretory vesicle
2	0030659	5.41	3.13	Cytoplasmic vesicle membrane
3	0030141	5.16	2.97	Secretory granule
4	0012506	5.06	3.04	Vesicle membrane
5	0030667	2.03	2.96	Secretory granule membrane

Cluster 05: Intrinsic component of presynaptic membrane

	Gene Ontology (GO)	Log10 (p-value)	Fold Enrichment (FE)	Cellular Component Annotation name
1	0098889	6.91	9.52	Intrinsic component of presynaptic membrane
2	0099240	6.55	6.42	Intrinsic component of synaptic membrane
3	0042734	5.99	6.21	Presynaptic membrane
4	0099699	4.80	5.63	Integral component of synaptic membrane
5	0099056	4.69	8.00	Integral component of presynaptic membrane
6	0099055	3.87	5.70	Integral component of postsynaptic membrane
7	0098936	3.72	5.46	Intrinsic component of postsynaptic membrane
8	0098945	3.29	14.03	Intrinsic component of presynaptic active zone membrane
9	0048787	2.47	8.60	Presynaptic active zone membrane
10	0099060	2.41	5.40	Integral component of postsynaptic specialization membrane
11	0098948	2.33	5.19	Intrinsic component of postsynaptic specialization membrane
12	0099634	2.32	4.57	Postsynaptic specialization membrane

Cluster 06: Intrinsic component of plasma membrane

	Gene Ontology (GO)	Log10 (p-value)	Fold Enrichment (FE)	Cellular Component Annotation name
1	0031226	6.46	2.59	Intrinsic component of plasma membrane
2	0005887	4.84	2.42	Integral component of plasma membrane

Cluster 07: Cytoplasmic region

	Gene Ontology (GO)	Log10 (p-value)	Fold Enrichment (FE)	Cellular Component Annotation name
1	0099568	4.78	3.48	Cytoplasmic region
2	0032838	4.31	4.76	Plasma membrane bounded cell projection cytoplasm
3	0120111	3.63	6.58	Neuron projection cytoplasm
4	1904115	3.16	7.55	Axon cytoplasm

Cluster 08: GABA-ergic synapse

	Gene Ontology (GO)	Log10 (p-value)	Fold Enrichment (FE)	Cellular Component Annotation name
1	0098982	3.86	7.11	GABA-ergic synapse

Cluster 09: Ion channel complex

	Gene Ontology (GO)	Log10 (p-value)	Fold Enrichment (FE)	Cellular Component Annotation name
1	0034702	3.84	3.78	Ion channel complex
2	1990351	3.80	3.61	Transporter complex
3	1902495	3.45	3.50	Transmembrane transporter complex
4	0034703	2.75	3.65	Cation channel complex
5	0098796	2.03	2.08	Membrane protein complex
6	0034704	2.02	5.21	Calcium channel complex

Cluster 10: Anchored component of plasma membrane

	Gene Ontology (GO)	Log10 (p-value)	Fold Enrichment (FE)	Cellular Component Annotation name
1	0046658	3.78	8.04	Anchored component of plasma membrane
2	0031225	2.15	3.59	Anchored component of membrane

Supplementary table 5. Specific genes related to the cellular components linked to the upper bound of the probability distribution.

Cluster 01: Ion channel complex	Cluster 02: Integral component of plasma membrane	Cluster 03: Synapse	Cluster 04: GABA-ergic synapse	Cluster 05: Cell junction
GABRA1	ATP2A2	ANK1	ATP2B2	ATP2A2
GABRB2	ATP2B2	ATP2A2	GABRA1	ATP2B2
GABRD	ATP4A	ATP2B2	GABRB2	CDH7
GABRG2	CRHR2	CRHR2	GABRD	EPB41
GABRG3	EDNRA	SLC29A1	GABRG2	GABRA1
GLRB	SLC29A1	EPB41	GABRG3	GABRB2
GRIN2A	FLT3	GABRA1	GLRB	GABRD
KCNA1		GABRB2	SLITRK3	GABRG2
KCNA2		GABRD	PLCB1	GABRG3
KCNB1		GABRG2	SLC6A17	GLRB
KCNC1		GABRG3		GRIN2A
KCNC3		GLRB		ICA1
KCNS1		GRIN2A		
PKD1		ICA1		
SCN1A		IL1RAP		
SCN1B				
SCN8A				
ABCC8				
TRPC3				
CACNG2				
LRRC8C				
LRRC38				
HCN1				
VWC2				

Supplementary table 6. Specific genes related to the cellular components linked to the lower bound of the probability distribution.

Cluster 01: Neuron projection	Cluster 02: Synapse	Cluster 03: Presynapse	Cluster 04: Secretory vesicle	Cluster 05: Intrinsic component of presynaptic membrane
APOE	APOE	ATP2B4	ANXA11	ATP2B4
RHOC	ATP2B4	CALB2	CD63	CDH9
ATP2B4	CALB2	CDH9	CDC42	CDH10
CALB2	CDC42	CDH10	CFD	CNR1
CAMK2D	CDH9	AP1S1	DPYSL3	EFNB2
CDC42	CDH10	CNR1	FABP5	GPC4
AP1S1	AP1S1	EFNB2	GABRA2	GABRA5
AP3S1	CNN3	GPC4	GRIA1	HTR3A
CNN3	CNR1	GABRA2	CXCL1	OPRK1
CNR1	DPYSL3	GABRA5	LOXL1	PCDH17
DMWD	EFNB2	GRIA1	MGST1	NTNG2
DPYSL3	FABP5	HTR3A	MIF	CPLX3
GABRA2	GPC4	NTSR1	NDUFC2	
GABRA3	GABRA2	OPRK1	HN1	
GABRA5	GABRA3	PDE2A	OPRK1	
GABRB1	GABRA5	PDYN	PCSK5	
GLRA2	GABRB1	PRKCG	PDGFB	
GNAO1	GLRA2	RAB27B	PRKCD	
GRIA1	GRIA1	SNCA	MAPK1	
GRM1	GRM1	DOC2A	KLK7	
HRH1	GUCY1A3	CASK	PYGL	
HTR1A	HTR3A	SNAP29	RAB27B	
HTR2C	NTSR1	SCGN	RAP2B	
HTR3A	OPRK1	C1QL1	SNCA	
KCNN3	PDE2A	PCDH17	SPARC	
KCNQ2	PDYN	SYT17	TIMP1	
NCAM2	PRKCG	SVOP	TIMP2	
NFIB	MAPK1	DTNBP1	TMSB4X	
NOV	PTPRA	NTNG2	DOC2A	
NTSR1	RAB27B	KCTD12	RNASET2	
OPRK1	SNCA	SYNPR	DYNLL1	
PDYN	SPARC	SLC17A8	SNAP29	
PRKCG	DOC2A	CPLX3	CAP1	
MAPK1	CASK		PRDX4	
PTPRF	SNAP29		PGRMC1	
RAB27B	MAPK8IP1		C9orf9	
ROBO1	CACNG3		MOXD1	
ROBO2	SCGN		ROBLD3	
SCN9A	PDLIM5		SYT17	
SLC1A4	RGS14		TRIM36	
SNCA	PGRMC1		SVOP	
SNCG	C1QL1		NIT2	
TIMP2	RAPGEF4		DTNBP1	
UCHL1	CORO1A		SYTL5	
DOC2A	FAM107A		LYZL4	
DYNLL1	LPHN3		SYNPR	
CPNE6	PSD3		TUBB	
MAPK8IP1	NPTXR		SLC17A8	
FAM65B	CADM1		CPLX3	
ODZ3	FBXO2			
CACNG3	DISC1			
SCGN	PCDH17			
PDLIM5	DACT1			
RGS14	SYT17			
PGRMC1	SVOP			
C1QL1	PCDH15			
CORO1A	TMEM108			
FAM107A	CAMKV			
KIF21B	GPR98			

LPHN3	DTNBP1
NPTXR	NTNG2
FBXO2	KCTD12
EXOC6	SNX26
NECAB2	SYNPR
KLHL1	SLC17A8
PCDH15	MDGA1
TMEM108	CPLX3
GPR98	
DTNBP1	
NTNG2	
SNX26	
UHMK1	
SYNPR	
PACRG	
SLC17A8	
CPLX3	

Supplementary table 7. Original curve fitting model analysis results and permutation analysis results. The regression analysis was performed between the mean dN/dS ratio and the evolutionary divergence times of the *Homo Sapiens*. The mean dN/dS ratio is an evolutionary measurement that captures the rate in which biological features are conserved in the species during evolutionary adaptive periods. The mean dN/dS ratios were obtained for each cellular component (*i.e.,* products of the genes with significant cortical expression within the phenotypic segregation-integration map) in the homologue-orthologue comparison between humans *vs.* non-human primates (*i.e.,* the chimpanzee, the gorilla, the orangutan, the macaque, the olive baboon, the vervet AGM, and the marmoset). Then, the mean dN/dS ratios were used to investigate the relationship between the S-I connectivity map and the *Homo Sapiens* evolutionary pathway. The cellular components linked to the upper bound of the probability distribution appear in Section A, and in Section B the ones linked to the lower bound of the probability distribution. The table contains the original curve fitting results (R^2, F-statistic and *p*-value), and the corrected *p*-value was obtained from the permutation analysis, which was done for testing the robustness of the original analysis.

Section A

	**Original analysis results**			**Permutation analysis results**
**Cellular components**	**R^2 value**	**F-value**	***p*-value**	**Corrected *p*-value**
Ion channel complex	0.880	14.73	0.01	0.001
Integral component of plasma membrane	0.767	6.600	0.05	<0.001
Synapse	0.332	2.489	0.18	-
GABA-ergic synapse	0.325	2.407	0.18	-
Cell junction	0.284	1.979	0.22	-

Section B

	**Original analysis results**			**Permutation analysis results**
**Celullar components**	**R^2 value**	**F-value**	***p*-value**	**Corrected *p*-value**
Neuron projection	0.861	30.99	0.003	<0.001
Synapse	0.859	30.53	0.003	0.004
Presynapse	0.617	8.04	0.036	0.407
Secretory vesicle	0.647	9.16	0.029	0.388
Intrinsic component of presynaptic membrane	0.390	18.7	0.019	0.498

2. SUPPLEMENTARY FIGURES

Supplementary Figure 1. Validation of the main connectomics results. The aim of the analysis was to test the robustness of the connectomics results by modifying different thresholds for the multiple-comparison correction. Brain maps appearing on the upper row were obtained after applying a logarithmic fit, which correspond to the *early trajectory mergers*. Similarly, on the lower row are showed the brain maps resulting after applying an exponential fit, which correspond to the *late trajectory mergers*. From left to right, the projections correspond to: uncorrected results; FDR-correction $q<0.05$; FDR-correction $q<0.001$; and, FDR-correction $q<0.0001$. **Note:** Brain maps are projected using CARET software. The color scale represents the 2-98% of the normalized connectivity data. **Abbreviations:** R = right hemisphere.

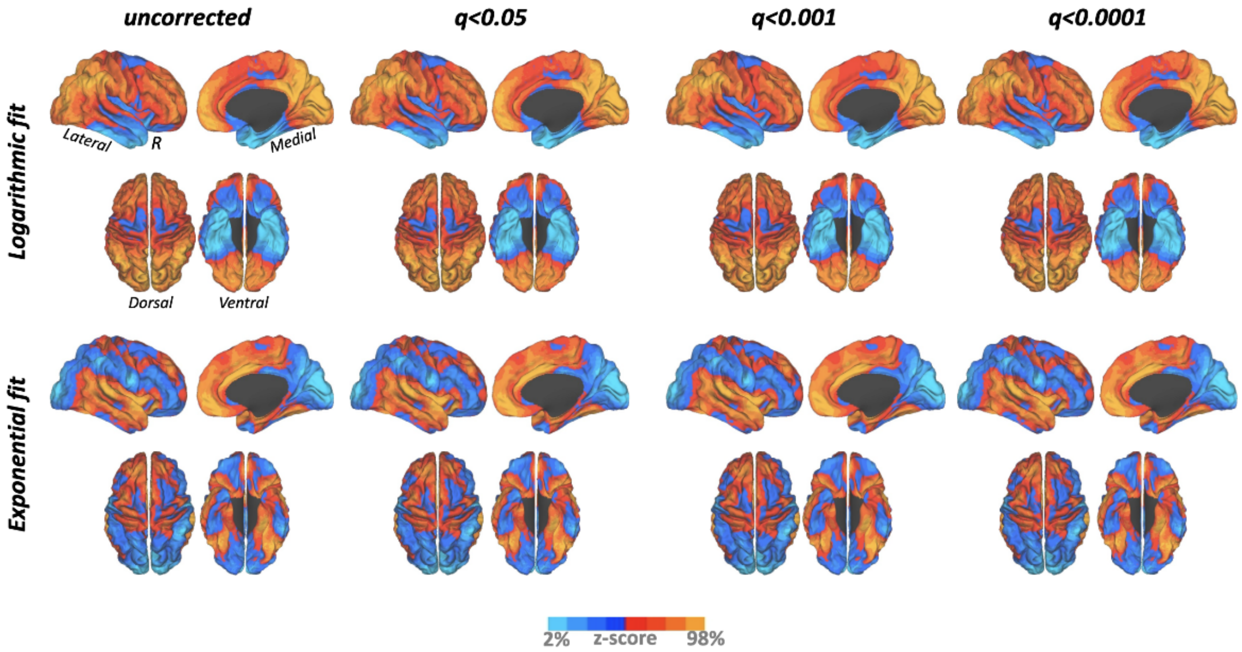

Supplementary Figure 2. Replication of the main connectomics results. The aim of the analysis was to test the robustness of the connectomics results using the resting-state functional MRI data of an independent sample. The results corresponding to the original sample appear on the left, while the results of the replication sample appear on the right. The brain maps related to the early trajectory mergers appear for both samples on the upper row, while the late trajectory mergers maps appear on the lower row. **Note:** Brain maps are projected using CARET software. The color scale represents the 2-98% of the normalized connectivity data. **Abbreviations:** L=left hemisphere; R = right hemisphere.

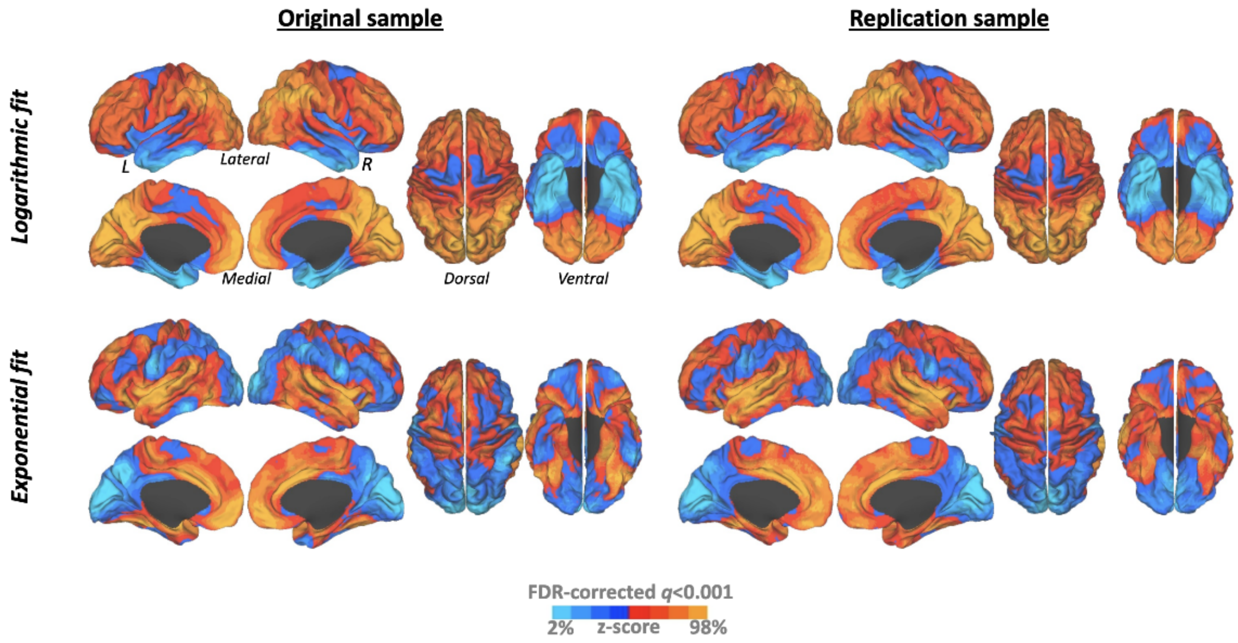

Supplementary Figure 3. Illustration of the merging process. The first row represents the nodes that are being progressively merged, while in the second row the links connecting the nodes have been plotted (upper 10% links). The left panels represent each node positioned in its MNI coordinate (upper) and the links connecting the nodes (lower). The middle and right panels represent two different moments of the merging process: two nodes were merged when they have similar connectivity and the MNI coordinates of the node with highest weighted degree was used to position "merged nodes" in the brain space. In this figure, node size represents the number of nodes that have been merged, thus, the more nodes where merged, the bigger the node size plotted. The original color palette used in Yeo et al. (Yeo et al. 2011, *J Neurophysiol, 106*(3):1125-65) was adopted in this illustration, therefore, nodes could be associated with known functional connectivity human brain networks. **Abbreviations:** Inf-Sup: inferior – superior axis; Ant-Post: anterior – posterior axis.

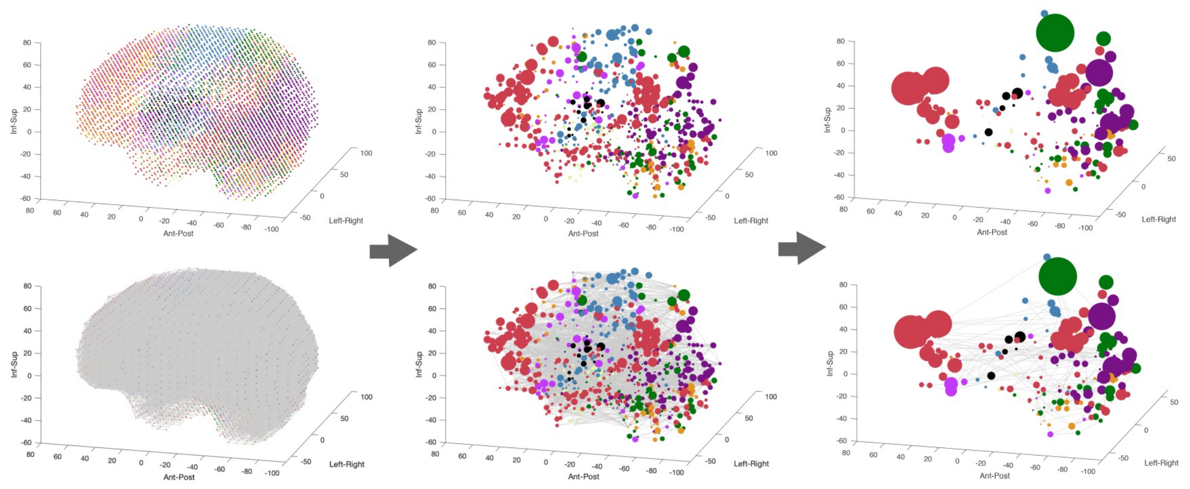

Supplementary Figure 4. Comparison of the merging process to a random assignation process. The left panel of the figure represents the normalized cumulative links using our merging rule, while the right panel represents the results of the process using a random merging rule. In this merging process, implementing our merging rule results in merging first segregated connections, while the integration is more predominant towards the end of merging process. Contrarily, using a random merging rule to classify the links leads to indistinctive assignations to segregation or integration. The Y axis represents the cumulative number of links, which has been normalized to account for the different number of links in segregation and integration. The X axis represents the merging time.

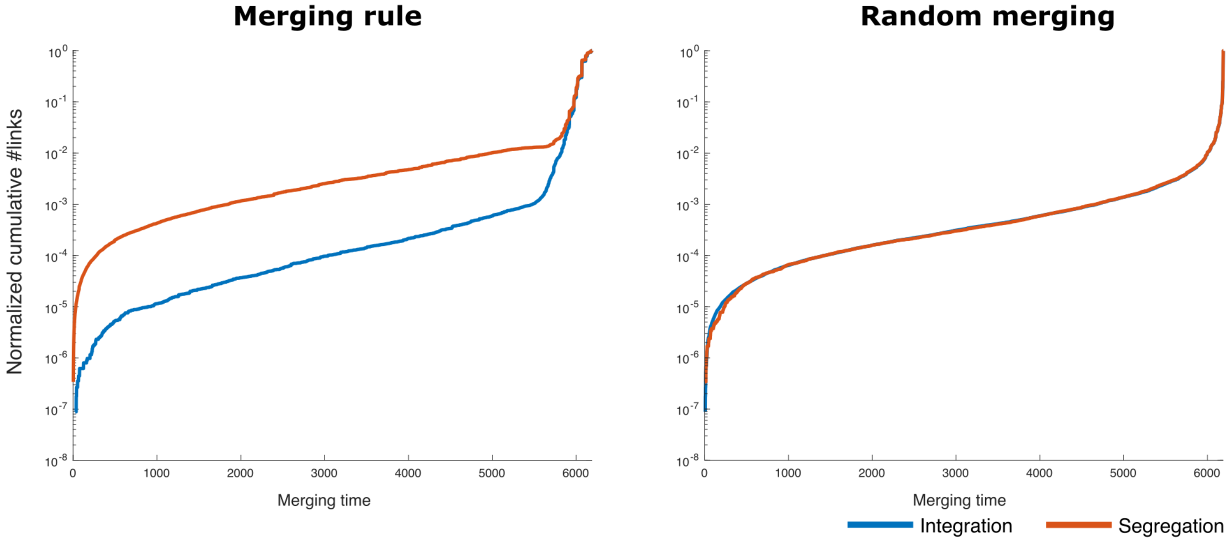

Supplement: Supplementary file 1 — Supplementary Information. [file 41598_2021_99082_MOESM1_ESM.pdf]
